# ASO Author Reflections: Redefining Prognostic Endpoints in Intrahepatic Cholangiocarcinoma—The Role of Recurrence-Free Survival

**DOI:** 10.1245/s10434-025-17212-0

**Published:** 2025-03-15

**Authors:** Jun Kawashima, Miho Akabane, Timothy M. Pawlik

**Affiliations:** 1 Department of Surgery, The Ohio State University Wexner Medical Center and James Comprehensive Cancer Center, Columbus, OH USA; 2 Department of Gastroenterological Surgery, Yokohama City University School of Medicine, Yokohama, Japan

## Past

Given the poor prognosis following curative-intent resection for intrahepatic cholangiocarcinoma (ICC), optimizing treatment strategies through multidisciplinary approaches is important.^1,2^ To date, several randomized controlled trials (RCTs) have been conducted in patients with biliary tract cancers (BTCs), including ICC, to evaluate the potential benefits of perioperative adjuvant therapies.^3^ In these studies, overall survival (OS) has traditionally been used as the primary endpoint, as it is considered the gold standard in oncology trials.^3^ However, reliance on OS presents notable limitations, requiring larger patient cohorts and prolonged follow-up, which can delay the clinical application of novel therapies.^3^ Consequently, there is an increasing need for surrogate endpoints that are both statistically robust and clinically meaningful, particularly for rare malignancies such as ICC.^4^ Recurrence-free survival (RFS) has emerged as a promising alternative, offering advantages such as shorter follow-up and faster trial completion. However, its validation as a surrogate for OS in ICC remains limited. Therefore, the current study aimed to evaluate the correlation between RFS and OS following surgical resection for ICC.

## Present

In the current study, we analyzed 1591 patients who underwent upfront curative-intent resection for ICC at multiple international institutions. The median RFS and OS were 22.6 months and 41.5 months, respectively. Notably, the correlation coefficient (*ρ*) between RFS and OS was 0.79 [95% confidence interval (CI) 0.76–0.82], indicating a moderately strong correlation. This association remained consistent among patients who received adjuvant chemotherapy (*ρ* = 0.78, 95% CI 0.72–0.82). Furthermore, in the landmark analysis, the concordance between 5-year survival in patients without recurrence and 5-year mortality among individuals with recurrence at different time points (6, 12, 24, 36, 48, and 54 months) was 60.7%, 72.0%, 81.4%, 83.1%, 83.0%, and 82.5%, respectively. These findings suggest that 3-year RFS may serve as a reliable surrogate endpoint for 5-year OS for patients with ICC.

## Future

To date, growing evidence suggests that ICC has a distinct biological profile compared with other BTCs, underscoring the necessity of ICC-specific clinical trials.^5^ Despite this need, ICC is a relatively rare disease, and most patients present with unresectable tumors at diagnosis. As a result, conducting perioperative adjuvant therapy trials with OS as the primary endpoint poses significant challenges owing to limited statistical power. Beyond adjuvant chemotherapy, there is increasing interest in neoadjuvant chemotherapy, targeted therapy, and immunotherapy for ICC.^2^ Further refinements in surgical management, combined with advancements in locoregional treatments and novel systemic therapies, are expected to improve patient outcomes. Given these evolving treatment paradigms, our study strongly supports the use of RFS as a reliable surrogate endpoint for OS in perioperative clinical trials involving patients undergoing surgery for ICC. This approach may enable more efficient trial designs and accelerate the development of effective treatment strategies for ICC.
